# Pars plana vitrectomy with partial tamponade of filtered air in Rhegmatogenous retinal detachment caused by superior retinal breaks

**DOI:** 10.1186/s12886-017-0459-6

**Published:** 2017-05-12

**Authors:** Zhaotian Zhang, Manjuan Peng, Yantao Wei, Xintong Jiang, Shaochong Zhang

**Affiliations:** State Key Laboratory of Ophthalmology, Zhongshan Ophthalmic Center, Sun Yat-sen University, 54S Xianlie Road, Guangzhou, 510060 China

**Keywords:** Air, Gas, Pars plana vitrectomy, Rhegmatogenous retinal detachment, Superior retinal break

## Abstract

**Background:**

To investigate the anatomic and functional outcomes of pars plana vitrectomy (PPV) with partial tamponade of filtered air for rhegmatogenous retinal detachment (RRD) caused by superior retinal breaks.

**Methods:**

Retrospective, comparative, consecutive case series study. Patients with RRD caused by superior retinal breaks undergone PPV with partial tamponade (Group A) and whole tamponade (Group B) of filtered air were included. The main outcomes were primary and final success rates, best corrected visual acuity (BCVA), and rate of postoperative cataract surgery.

**Results:**

Forty-one patients (41 eyes) were included in Group A and 36 patients (36 eyes) were included in Group B. There were no significant differences in primary or final success rates between Groups A and B (*P* = 0.618 and *P* = 1.000, respectively). The patients in Group A experienced quicker postoperative vision improvement (from the Week 1 follow-up) than the patients in Group B (from the Month 3 follow-up). The postoperative cataract surgery rate of Group A (7/31) was lower than that of Group B (13/26) (*P* = 0.031).

**Conclusions:**

PPV with partial tamponade of air is effective in achieving a high anatomic success rate, quicker postoperative vision improvement, and lower rate of postoperative cataract surgery in RRD caused by superior retinal breaks.

## Background

Rhegmatogenous retinal detachment (RRD) refers to retinal breaks accompanied by subretinal fluid accumulation through the primary retinal breaks. Pars plana vitrectomy (PPV) is becoming increasingly popular for the treatment of RRD, due to the ease of application and the satisfactory visualization of the peripheral retina with scleral indentation and wide-angle viewing systems [1–4]. RRD caused by superior retinal breaks puts patients in an urgent condition in which prompt surgical intervention is necessary to prevent rapid vision deterioration. After removal of the vitreous, various endotamponade agents, such as air, long-acting gas, and silicone oil, are injected into the vitreous cavity to generate surface tension across the retinal breaks to strengthen retinal reattachment until the retinopexy becomes mature [2, 5, 6].

Considering the potential risks of silicone oil tamponade and the necessity of secondary removal, the tendency is to use gas tamponade for the repair of RRD caused by superior retinal breaks without giant retinal tears or severe proliferative vitreoretinopathy [7–10]. Previous studies have suggested that the outcomes of air tamponade used in treating RRD were comparable with those of long-acting gas tamponade, which is consistent with our clinical observations [8, 11, 12]. However, we have noticed that intraocular gas poses some problems. For example, eyes that received gas tamponade had blurred vision from refractive changes until the gas bubble was absorbed to be above the visual axis, which was very inconvenient for patients with poor visual acuity of the contralateral eye [7]. In addition, the contact between the gas and the posterior lens capsular could result in an increased chance or early onset of nuclear sclerotic cataract formation [13–15].As such, we tried to provide partial fluid/air exchange at the end of PPV for the repair of RRD caused by superior retinal breaks, in order to shorten the time of visual rehabilitation and ensure that the visual axis was transparent, for better visual acuity several days after surgery. A thorough search of the literature revealed no recent reports on partial air tamponade used in the repair of RRD caused by superior retinal breaks. Therefore, this retrospective study was conducted to compare the long-term outcomes of partial air tamponade and whole air tamponade for the repair of RRD caused by superior retinal breaks.

## Methods

The medical records of all patients at Zhongshan Ophthalmic Center who underwent 23/25-gauge PPV with filtered air tamponade (partial or whole) from May 1, 2014 to September 1, 2015 to treat RRD caused by superior retinal breaks were reviewed retrospectively, and written consent for surgical treatment had been obtained from each subject. The inclusion criteria were eyes with RRD caused by superior retinal breaks located above the horizontal meridian (9–3 o’clock) that underwent primary 23/25-gauge PPV with filtered air tamponade (partial or whole). Patients with RRD caused by superior retinal breaks undergone PPV with partial tamponade were categorized in Group A; and patients with RRD caused by superior retinal breaks undergone PPV with whole tamponade categorized in Group B. The exclusion criteria were previous vitreoretinal surgery; significant subcapsular opacity; follow-up of less than six months; other serious eye diseases; and incomplete data. Written informed consent was obtained from each patient. The main outcomes were primary and final anatomic success rates, best corrected visual acuity (BCVA), and rate of postoperative cataract surgery. Secondary outcomes were intraoperative and postoperative complications.

All eligible patients underwent comprehensive ophthalmologic examinations during the follow-up period, including Snellen BCVA, non-contact tonometry, slit-lamp microscopy, dilated funduscopic examination, and assessment of lens status. Additional data collected includes gender, disease course, range of retinal detachment, and number and area of retinal breaks. During the surgical procedure, the peripheral retina was checked repeatedly for any undetected retinal breaks, and degenerative areas, the presence of posterior vitreous detachment, the use of heavy liquid (perfluoro-n-octane), and intraoperative complications were also recorded.

All of the surgeries were performed under retrobulbar anesthesia by one experienced surgeon (S.Z.). Patients who had cataracts with visual significance (all patients were assessed by S.Z.) were required to have concurrent cataract extraction and intraocular lens (IOL) implantation. A standard three-port, 23- or 25-gauge pars plana vitrectomy (Constellation Vitrectomy System; Alcon Laboratories, Fort Worth, TX) was performed. First, a core vitrectomy was performed and the peripheral vitreous was further cut off, using a wide-angle viewing system and scleral indentation. The peripheral retina was inspected repeatedly to determine whether there were any retinal breaks or degenerative areas. The subretinal fluid was drained off using aspiration of the vitrectomy probe and flute needle. If the detached retina could not be flattened well after complete vitrectomy and fluid/air exchanges, heavy liquid (which was completely drained off with fluid/air exchange after retinopexy) was used in addition. Endolaser photocoagulation was applied three to five rows around to seal the retinal breaks and degenerative areas. After complete retinopexy was achieved, partial or whole fluid/air exchange was performed in order to have drainage of the balanced salt solution. In patients who received partial air tamponade, the surgeon paid special attention to the amount of fluid/air exchange based on the locations and areas of the superior retinal breaks. Finally, all the cases in Group A underwent 50% tamponade of filtered air at the conclusion of surgery.

After surgery, all of the patients were instructed to remain in semi-recumbent position at least 12 h/day for three days. Each patient’s head position was adjusted slightly, according to the accurate location of the superior retinal breaks. On the first day after surgery, the patients were examined for postoperative complications. Patients with no serious postoperative complications were discharged one day after undergoing surgery. Follow-up examinations were scheduled at one week and one, three, and six months after surgery. Extra visits were scheduled as needed. During the postoperative follow-ups, it was recommended that all phakic eyes with new-onset or deterioration of lens opacification should have cataract extraction and IOL implantation once there was visual significance (at least two months after the RRD repair surgery and one month prior to the last follow-up visit). Pseudophakic eyes were recommended for neodymium-doped yttrium aluminum garnet (Nd: YAG) laser subcapsulotomy if the subcapsular opacity was visually significant. Anatomic success was defined as the complete disappearance of subretinal fluid and complete attachment of the borders of the neuroretina to the underlying retinal pigment epithelium by retinopexy.

All Snellen visual acuity values were converted to the logarithm of the minimum angle of resolution (logMAR) for statistical analysis. Visual acuity of light perception was assigned 2.9, hand movements 2.6, and counting fingers 2.3 [16]. All data were analyzed using SPSS 19.0 statistical software (SPSS Inc., Chicago, IL). Paired/unpaired *t* tests, paired/unpaired Mann–Whitney tests, and χ^2^ analysis were used as appropriate. All continuous data were expressed as mean ± standard deviation. A *P* value less than 0.05 was considered statistically significant.

## Results

Seventy-seven patients (77 eyes) were included in this study, with 41 patients (41 eyes) in Group A and 36 patients (36 eyes) in Group B.

There were no significant differences in age, gender, disease course, follow-up duration, preoperative logMAR BCVA, or other preoperative intraocular parameters between Groups A and B (all *P* > 0.05). Sixteen patients in Group A had low vision (Snellen BCVA <20/200) of the contralateral eye, more than in Group B (*P* = 0.013). Table 1 shows the baseline characteristics of all of the subjects in Groups A and B.

**Table 1:** Baseline characteristics of patients in Group A (*n* = 41 eyes) and Group B (*n* = 36 eyes)

	Group A	Group B	*P* value
Gender (male: female)	28: 13	27: 9	0.516^*^
Age (years)	49.2 ± 12.9	47.4 ± 11.6	0.535^†^
≤ 50 (number)	21	19	0.891^*^
> 50 (number)	20	17	
Contralateral eyes with BCVA <20/200 (number)	16	5	0.013^*^
Disease course (days)	15.0 ± 8.7	11.94 ± 7.2	0.098^†^
Follow-up duration (months)	12.9 ± 4.5	13.6 ± 4.7	0.473^†^
Preoperative logMAR BCVA (Snellen equivalent)	1.22 ± 0.64 (20/332)	1.12 ± 0.58 (20/264)	0.496^†^
Range of RD (clock hours)	4.7 ± 1.5	4.6 ± 1.3	0.707^†^
Macula (on: off)	23: 18	22: 14	0.656^*^
Superior retinal breaks			
Number	1.9 ± 1.0	2.1 ± 1.0	0.359^‡^
Total area (PD)	2.1 ± 1.0	2.2 ± 1.0	0.646^†^
Lens status (phakic: pseudophakic)	35: 6	31: 5	0.926^*^
Presence of Inferior retinal breaks (yes: no)	11: 30	10: 26	0.926^*^

*BCVA* best corrected visual acuity, *logMAR* logarithm of minimal angle of resolution, *RD* retinal detachment, *PD* papilla diameter

Statistical analysis: * = χ^2^ analysis; ^†^ = unpaired *t* test; ^‡^ = unpaired *Mann-Whitney* test

Continuous values presented as mean ± standard deviation,

There were no significant differences in the intraoperative use of heavy liquid or endolaser coagulation between Groups A and B (*P* = 0.675 and *P* = 0.715, respectively). The rates of phacovitrectomy in Groups A and B were 9.8% (4/41) and 13.9% (5/36), respectively (*P* = 0.726). The primary anatomic success rates in Groups A and B were comparable; there was no significant difference (*P* = 0.618). A final success rate of 100% was achieved in both groups, without secondary scleral buckling or silicone oil tamponade. The intraoperative and postoperative parameters of all of the patients are presented in Table 2.

**Table 2:** Intraoperative and postoperative parameters collected from all patients in Group A (*n* = 41 eyes) and Group B (*n* = 36 eyes)

	Group A	Group B	*P* value
Use of heavy liquid (yes:no)	29:12	27:9	0.675^*^
Phacovitrectomy (yes:no)	4:37	5:31	0.726^*^
Laser photocoagulation number (shots)	421.3 ± 131.5	450.9 ± 150.3	0.715^†^
Primary anatomic success (yes:no [%])	38:3 (92.7)	35:1 (97.2)	0.618^*^
Causes of failure (number of eyes)			1.000^*^
New retinal breaks	2	1	
PVR	1	0	
Final anatomic success (%)	100	100	1.000^*^
Postoperative cataract surgery (number)			
Phakic eyes after primary RD repair	31	26	0.735^*^
Underwent secondary cataract surgery	7	13	0.031^*^
Remained phakic until the final follow-up	24	13	0.049^*^
Eyes underwent Nd: YAG subcapsulotomy	4	12	0.068^*^
Postoperative interval to have cataract surgery (months)	10.3 ± 3.0	9.9 ± 2.9	0.797^‡^
Postoperative logMAR BCVA (Snellen equivalent)			
Day 1	1.41 ± 0.60 (20/514)	2.45 ± 0.23 (20/5637)	<0.001^†^
Week 1	0.96 ± 0.51 (20/182)	1.18 ± 0.40 (20/303)	0.008^‡^
Month 1	0.86 ± 0.37 (20/145)	1.08 ± 0.29 (20/240)	0.003^‡^
Month 3	0.72 ± 0.39 (20/105)	0.79 ± 0.30 (20/123)	0.182^‡^
Month 6	0.63 ± 0.34 (20/85)	0.61 ± 0.27 (20/81)	0.769^†^
Final follow-up	0.42 ± 0.30 (20/53)	0.43 ± 0.21 (20/54)	0.404^‡^

*PVR* proliferative vitreoretinopathy, *RD* retinal detachment, *Nd: YAG* neodymium-doped yttrium aluminum garnet, *logMAR* logarithm of minimal angle of resolution, *BCVA* best corrected visual acuity

Statistical analysis: * = χ2 analysis; ^†^ = unpaired *t* test; ^‡^ = unpaired *Mann-Whitney* test

Continuous values presented as mean ± standard deviation

There was no significant difference in the rate of phakic eyes between Groups A and B (75.6% [31/41] versus 72.2% [26/36]; *P* = 0.735) immediately after the primary RD repair. Seven phakic eyes (22.6%) in Group A underwent secondary cataract surgery, whereas 13 eyes (50.0%) in Group B underwent secondary surgery, significantly more than in Group A (*P* = 0.031). During the follow-up period, four (23.5%) pseudophakic eyes in Group A and 12 (52.2%) pseudophakic eyes in Group B underwent Nd: YAG subcapsulotomy (*P* = 0.069).

**Fig. 1:**
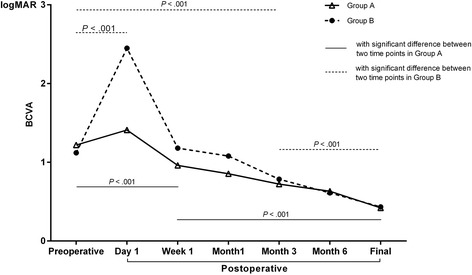
Change tendency of best corrected visual acuity (logMAR unit) at different time points in Group *A* and Group *B*

At the one-week follow-up, the patients in Group A began to show postoperative vision improvement compared with their preoperative logMAR BCVAs (*P* < 0.001), and their BCVAs were better at the final follow-up than at the three-month follow-up (0.42 ± 0.30 [20/53] versus 0.72 ± 0.39 [20/105]; *P* < 0.001). The BCVAs of the patients in Group B decreased sharply from the preoperative measurements to postoperative Day 1 (*P* < 0.001), but they began to show improvement at the three-month follow-up (*P* < 0.001). Postoperative BCVAs were better in Group A than in Group B at the Day 1, Week 1, and Month 1 follow-ups (*P* < 0.001, *P* = 0.080, and *P* = 0.003, respectively). There were no significant differences in logMAR BCVAs at the three-month follow-up between Groups A and B (all *P* > 0.05). The change tendencies of the logMAR BCVAs of Group A and Group B are displayed in Fig. 1.

**Fig. 2:**
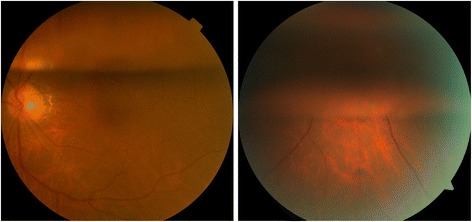
Typical fundus images of patients included in the study on postoperative Day 1. Group A (*left*): the air bubble remained at the top one-third of the vitreous cavity, with transparency of the visual axis. Group B (*right*): the air bubble remained at the top three-fifths of the vitreous cavity, without transparency of the visual axis

At the postoperative Day 1 examination, it was observed that air filled the upper vitreous cavity (the height of the air bubbles ranged from one-fifth to one-third of the height of the vitreous cavity) of all the operated eyes in Group A, with transparency of the visual axis. In addition, the operated eyes in Group B were all filled with air bubbles at the upper 1/2–4/5 of the vitreous cavity. Fig. 2 shows typical fundus images of Group A and Group B on the first day after surgery. No serious intraoperative or postoperative complications were observed, and no eyes experienced hypotony (<5 mmHg) before or after the surgery. Temporary intraocular pressure elevation (>21 mmHg) after the surgery was controlled well and brought back to the normal range with the topical administration of eye drops.

## Discussion

Gas tamponade provides surface tension and buoyancy against superior retinal breaks, thereby preventing fluid accumulation under the subretinal space while the retinopexy matures [5, 7]. Conventionally, long-acting gases such as octafluoropropane (C_3_F_8_) and sulfur hexafluoride (SF_6_) are commonly used in the repair of RRD and macular holes [1, 17–19]. However, the half-lives of long-acting gases are relatively long, and the presence of intraocular gas greatly affects the speed of postoperative visual rehabilitation after PPV. In addition, patients are required to maintain a prolonged face-down position for several days after surgery. Therefore, attempts have been made to shorten the time of visual rehabilitation with different types and concentrations of tamponade agents [5, 7–9, 16, 20]. Due to the shorter half-life and the nonexpansile property of intraocular air, there has been a trend to broaden its use in some vitreoretinal surgeries [7, 8, 20]. Tan et al. [8] reported that air tamponade should only be used in RD restricted to the superior quadrants, which is consistent with the inclusion criteria of the current study. To the contrary, a recent prospective study by Zhou et al. [20] suggested that air had tamponade effects equivalent to those of C_3_F_8_, with a shorter prone positioning period, in the repair of RRD with inferior breaks.

Unlike those previous studies, the major difference between Group A and Group B in the current study was the amount of air tamponaded in the vitreous cavity at the end of PPV. While air was theoretically present for a shorter time in Group A than in Group B, there were no significant differences in term of primary and final success rates between the two groups (*P* = 0.618 and *P* = 1.000, respectively). The high anatomic success rates indicate that air tamponade is enough to seal superior retinal breaks for the establishment of strong chorioretinal adhesion [7, 11]. The amount of air used in the tamponade did not act as a significant factor on the anatomic success rate of the repair of RRD caused by superior breaks. To the best of our knowledge, there are no other recent reports regarding the outcomes of PPV with partial air tamponade in the treatment of vitreoretinal diseases. The satisfactory anatomic outcomes might be due to the following five factors: first, complete vitreous shaving in the core and peripheral areas to interrupt the occurrence of PVR; second, the subretinal fluid was completely drained off in all operated eyes, which was important for successful retinopexy with endolaser photocoagulation; third, the ranges of RD (4.7 and 4.6 clock hours in Group A and Group B, respectively; *P* = 0.707) in both groups were relatively smaller without severe PVR; fourth, although the amount of intraocular air varied between Group A and Group B, the numbers and total areas of superior retinal breaks were not too large for the temporary air tamponade to support the reattached retina to the choroid without reoccurrence of subretinal fluid accumulation; and finally, the retina was thoroughly checked for any retinal breaks and degenerative areas, and the intraoperative interventions prevented secondary retinal detachment.

In addition to the anatomic success rates, significant improvements in BCVA were observed in both groups at the final follow-up visit compared with preoperative levels (both *P* < 0.001). There were no significant differences in logMAR BCVA between Group A and Group B in the preoperative and postoperative month 3, month 6, and final follow-up measurements (*P* = 0.496, *P* = 0.182, *P* = 0.769, and *P* = 0.404, respectively). The most remarkable difference in logMAR BCVA between the two groups was observed at the postoperative day 1 visit (1.41 ± 0.60 versus 2.45 ± 0.23, *P* < 0.001). The decrease in Snellen BCVA on the first day after surgery was much smaller in Group A (from 20/332 to 20/514) than in Group B. It was observed that the rate of patients with poor vision (<20/200) in the contralateral eye was higher in Group A (16/41) than in Group B (5/36), which was one of the driving factors to conduct the pilot trial of partial air tamponade at the end of PPV. Due to the sharp refractive changes induced by the intraocular air under the visual axis, all of the patients in Group B experienced blurred vision (all ≤20/200) on the first day after surgery. As we know, air bubble in the vitreous cavity cannot expand and exerts an effect for 5–7 days. We could conclude that the lower visual acuity of Group B in postoperative first month should be contributed by the longer contact of filtered air with the lens. The lens of patients in Group B was exposed to a higher oxygen tension, which prevented the quicker vision rehabilitation in Group B. Additionally, some cases with transient feathering of the posterior capsule might not have completely restored transparency in postoperative first month. Even there was low vision of Groups A and B in postoperative first month, we could see the tendency to have vision improvement in both groups was obvious.

Although not supported by objective data, we could assume that the sharp decrease in visual acuity after surgery would cause inconvenience and fear, especially in patients with poor visual acuity in the contralateral eye. The results suggest that one of the advantages of partial air tamponade for the repair of RRD caused by superior retinal breaks is to speed up visual rehabilitation after surgery.

Another advantage of partial air tamponade over whole air tamponade is the lower rate of postoperative cataract surgery: previous studies have reported an acceleration of nuclear sclerosis after lens-sparing vitrectomy [13–15, 21, 22]. It has also been found that intraocular gas can cause a temporary feathery posterior subcapsular opacity after surgery. Holekamp et al. [15] found that exposure of the crystalline lens to abnormally high oxygen levels can lead to nuclear cataract formation in vitrectomized eyes. However, due to the retrospective design of the current study and the clinical routine of our vitreoretinal clinic, we were unable to provide quantitative evaluations of lens opacity with the commonly used lens opacity classification systems. Instead, we chose the rate of postoperative cataract surgery to analyze the relationship between amount of intraocular air and formation of secondary cataract in this retrospective study. There were 31 phakic eyes in Group A and 26 in Group B immediately after the primary RD repair surgery (*P* = 0.735). During the follow-up visits, all of the phakic eyes were assessed with slit-lamp examination to determine whether cataracts of visual significance were present. The vitreoretinal surgeon (S.Z.) decided whether cataract surgery was necessary based on his clinical experience. The rate of postoperative cataract surgery was lower in Group A than in Group B (7/31 versus 13/26, *P* = 0.039), because phakic eyes in Group A with partial tamponade of air had shorter duration of intraocular air contacting with the lens. We could hypothesize that the lower oxygen tension exerting on the lens of Group A was the main reason for the lower rate of postoperative cataract surgery [14, 15]. However, the difference of rates of phakic eyes undergone Nd: YAG subcapsulotomy in Group A and B was not statistically significant (*P* = 0.068). Even though, we believe that the difference would become more significant if more cases were included in the study.

There were several concerns about the current method of partial air tamponade for repair of RRD caused by superior retinal breaks. The areas and locations of retinal detachment should be checked and analyzed meticulously before and during the surgery [23]. Otherwise, the relatively smaller amount of air might not act well to support the retinal breaks until the retinopexy becomes firm enough. As reported by Rahman, R. et al. [24], the core and peripheral vitreous should be cut off completely in order to interrupt the formation of PVR, but it might increase the possibility of iatrogenic retinal holes and lens contact. The subretinal fluid should be drained off completely to flatten the retina for better endolaser retinopexy. Finally, care should be taken to determine accurately the amount of air tamponaded, to ensure that all major retinal breaks are well sealed.

This study had several limitations. It was a retrospective study with a small sample size and short duration of follow-ups. In addition, we were unable to judge lens opacity based on an objective standard.

## Conclusions

In conclusion, PPV with partial air tamponade can be performed to achieve a high anatomic success rate in treating RRD caused by superior retinal breaks, with more rapid postoperative visual recovery and a lower rate of postoperative cataract surgery. Further randomized case–control studies with a larger sample size and an objective standard should be undertaken.

## Abbreviations

PPV: Pars plana vitrectomy
RRD: Rhegmatogenous retinal detachment
BCVA: Best corrected visual acuity
logMAR: Logarithm of the minimum angle of resolution
Nd;YAG: Neodymium-doped yttrium aluminum garnet
C_3_F_8:_: Octafluoropropane
SF_6_: Sulfur hexafluoride
RD: Retinal detachment
PVR: Proliferative vitreoretinopathy
